# Antimicrobial Property and Mode of Action of the Skin Peptides of the Sado Wrinkled Frog, *Glandirana susurra,* against Animal and Plant Pathogens

**DOI:** 10.3390/antibiotics9080457

**Published:** 2020-07-29

**Authors:** Daisuke Ogawa, Manami Suzuki, Yuriko Inamura, Kaito Saito, Itaru Hasunuma, Tetsuya Kobayashi, Sakae Kikuyama, Shawichi Iwamuro

**Affiliations:** 1 Department of Biology, Faculty of Science, Toho University, 2-2-1 Miyama, Funabashi, Chiba 274-8510, Japan; diceke.ogawa210714@gmail.com (D.O.); manami.ro52@gmail.com (M.S.); horizon.rosso@gmail.com (Y.I.); koumori88@ozzio.jp (K.S.); i.hasunuma@sci.toho-u.ac.jp (I.H.); 2 Department of Regulatory Biology, Faculty of Sciences, Saitama University, 255 Shimo-okubo, Sakura-ku, Saitama 338-8570, Japan; tkoba@mail.saitama-u.ac.jp; 3 Department of Biology, Faculty of Education and Integrated Arts and Sciences, Center for Advanced Biomedical Sciences, Waseda University, 2-2 Wakamatsu-cho, Shinjuku-ku, Tokyo 162-8480, Japan; kick-yama@waseda.jp

**Keywords:** Sado wrinkled frog, frog skin, antimicrobial peptides, anti-plant-pathogen activity, ELEBA

## Abstract

The Sado wrinkled frog *Glandirana susurra* has recently been classified as a new frog species endemic to Sado Island, Japan. In this study, we cloned 12 cDNAs encoding the biosynthetic precursors for brevinin-2SSa–2SSd, esculentin-2SSa, ranatuerin-2SSa, brevinin-1SSa–1SSd, granuliberin-SSa, and bradykinin-SSa from the skin of *G. susurra*. Among these antimicrobial peptides, we focused on brevinin-2SSb, ranatuerin-2SSa, and granuliberin-SSa, using their synthetic replicates to examine their activities against different reference strains of pathogenic microorganisms that infect animals and plants. In broth microdilution assays, brevinin-2SSb displayed antimicrobial activities against animal pathogens *Escherichia coli*, *Salmonella enterica*, *Pseudomonas aeruginosa*, and *Candida albicans* and plant pathogens *Xanthomonas oryzae* pv. *oryzae*, *Clavibacter michiganensis* subsp. *michiganensis*, and *Pyricularia oryzae*. Ranatuerin-2SSa and granuliberin-SSa were active against *C. albicans* and *C. michiganensis* subsp. *michiganensis*, and granuliberin-SSa also was active against the other plant pathogenic microbes. Scanning electron microscopic observations demonstrated that brevinin-2SSb, ranatuerin-2SSa, and granuliberin-SSa induced morphological abnormalities on the cell surface in a wide range of the reference pathogens. To assess the bacterial-endotoxin-binding ability of the peptides, we developed an enzyme-linked endotoxin-binding assay system and demonstrated that brevinin-2SSb and ranatuerin-2SSa both exhibited high affinity to lipopolysaccharide and moderate affinity to lipoteichoic acid.

## 1. Introduction

The increase in drug-resistant pathogenic microorganisms is a serious public health problem worldwide. For example, certain nosocomial pathogens are already resistant to major available antibiotics. Finding novel antibiotics is becoming more and more difficult. Accordingly, development of new types of antimicrobial agents possessing completely different antimicrobial mechanisms from conventional antibiotics is awaited [1]. Antimicrobial peptides (AMPs) are promising candidates to suppress such pathogens. AMPs are gene-encoded polypeptides of various lengths and structures that are found in all organisms, including vertebrates, invertebrates, plants, and bacteria [2]. Most AMPs contain cationic and hydrophobic amino acid residues that facilitate formation of amphipathic α-helical or β-sheet conformations in a membrane-mimetic environment [3]. Thus, AMPs are believed to be electrostatically attracted to negatively charged microbial surfaces and interact with the negatively charged external leaflet of the microbial cytoplasmic membrane, and AMPs may then adopt an amphipathic conformation, which allows the insertion of the hydrophobic face into the bilayer to form pores by “barrel-stave”, “carpet”, or “toroidal-pore” mechanisms [4]. Thus, in contrast to conventional antibiotic drugs, the antimicrobial activities of AMPs depend on their primary and secondary structures and can act against a broad range of microorganisms in a nonspecific manner [5,6]. Because of these characteristics, AMPs have been proposed as possible additives or replacements for conventional antibiotics. AMPs also appear to induce antibiotic resistance in some bacteria [7] but do so at rates much lower than those of conventional antibiotics. 

Amphibian skins are a rich source for AMPs because of the worldwide distribution of amphibians and their wide range of environmental habitats, occupying aquatic, semiaquatic, and/or terrestrial environments. These skins are the structural and functional interface between the organism and its environment. To defend themselves from invasion by a wide variety of microorganisms, amphibians have developed AMPs as an innate defense system. Currently, 1095 amphibian AMPs are found in the Antimicrobial Peptide Database 2020 (http://aps.unmc.edu/AP/). Based on limited amino acid sequence similarities, these AMPs can be classified into several groups: temporin, brevinin-1, brevinin-2, ranatuerin-2, esculentin-2, etc. [8,9]. However, their sequences are so hypervariable that AMPs possessing the same sequences are rarely found among different frog species. Additionally, the application of molecular techniques of phylogenetic analysis and comparison of mitochondrial nucleotide sequences has led to quite drastic reappraisals of taxonomic classifications and evolutionary histories of amphibians [10]. Many previous and well-accepted phylogenetic relationships that were based upon classical criteria, such as morphological characteristics and the fossil record, are being substantially revised. Therefore, a frog species not yet examined for AMPs may be a good source for novel AMPs. Thus, amphibians, especially those belonging to the family Ranidae and the genus *Rana*, which is one of the most diverse and worldwide amphibian groups, are preferably used for sources of AMPs. Ranid frog AMPs are produced from precursor proteins via specific enzymatic cleavage events. These precursors contain a signal peptide region, an intervening sequence region, and an AMP region. While nucleotide sequences of AMP coding regions show little similarity even among closely related species, the signal peptide regions are highly conserved [11]. Moreover, the 3′-untranslated regions (3′-UTRs) of AMP family precursor cDNAs comprise relatively specific sequences [12]. Taking advantage of these nucleotide sequence similarities, AMP precursor cDNAs have been cloned using reverse-transcriptase polymerase chain reaction (RT-PCR) and 3′ rapid amplification of cDNA ends (3′-RACE) [13].

The Japanese wrinkled frog has been believed to inhabit mainland Japan (except Hokkaido) and some adjacent islands such as Sado, Oki, Iki, Yaku, and Goto Island Group. However, Sekiya et al. [14,15] proposed some genetic and phylogenetic differentiation between the frog from Sado Island and the species inhabiting other Japanese locations. This frog has now been registered as a new species and named the Sado wrinkled frog. Based on the histories of the genera *Rugosa* and *Glandirana*, the Sado wrinkled frog was first named *Rugosa susurra* but has now been re-renamed as *Glandirana susurra* [16]. This small-sized anuran is distributed only on Sado Island, which lies off the coast of Niigata Prefecture, Japan. In the present study, we focused on this newly classified species, *G. susurra*, and cloned 12 cDNAs, encoding precursors for their AMPs by RT-PCR using sets of specific primers. Among these, we generated cDNA clones that encoded unique peptides and examined the antimicrobial and cytotoxic properties of synthetic replicates of these peptides, namely brevinin-2SSa, ranatuerin-2SSa, and granuliberin-SSa. The nomenclature used to describe these peptides is the same as that used for ranid frog skin peptides; the species “*susurra*” is indicated by “SS”, and isoforms are designated by lowercase letters [8].

## 2. Results

### 2.1. Nucleotide and Amino Acid Sequence Analyses

**Figure 1:**
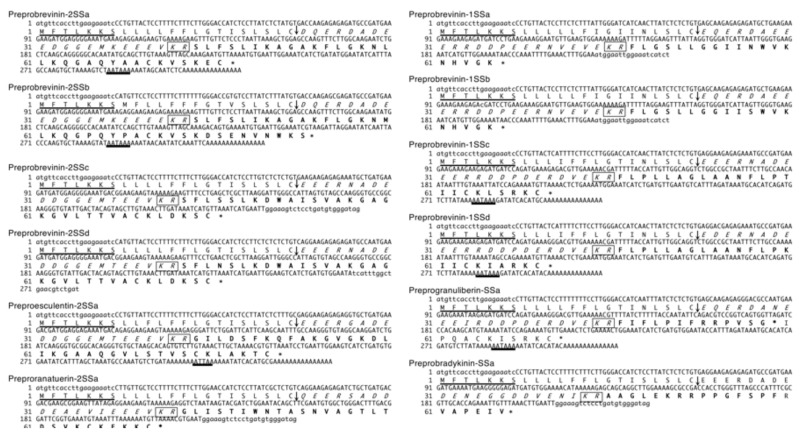
Nucleotide and deduced amino acid sequences of antimicrobial peptide (AMP) precursor cDNAs that were cloned from *Glandirana* *susurra* skin. In the nucleotide sequences, forward- and reverse-primer-derived sequences are presented in lowercase. In the amino acid sequences, forward-primer-derived sequences are underlined, intervening sequences are presented in italics, AMP sequences are presented in bold, stop codons are marked with asterisks (*), and cleavage sites for signal peptidase and processing enzymes are marked with arrows and boxes, respectively.

**Figure 2:**
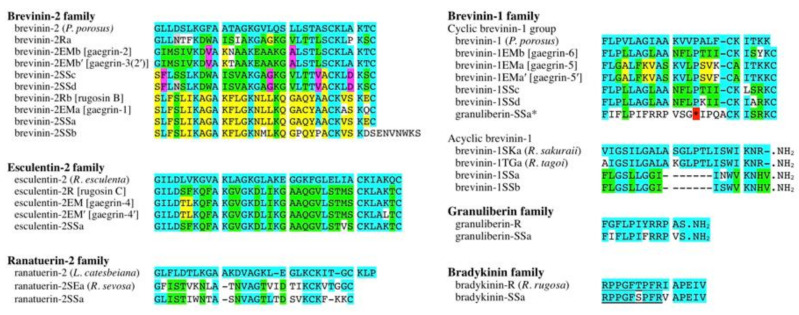
A comparison and schematic alignment of amino acid sequences of the brevinin-2, esculentin-2, ranaturerin-2, brevinin-1, granuliberin, and bradykinin peptides deduced from *G. susurra* AMP precursor homolog cDNAs. Residues conserved among the *Glandirana* peptides and orthologs from other species are marked by the same color. The reference sequences of each peptide family (mainly the originally isolated ones) are at very upper lines. Gaps (-) were introduced to maximize the sequence identities. The asterisk (*) indicates a stop codon in the granuliberin-SSa, and the sequence following the asterisk shows high similarities to that of the cyclic brevinin-1SS peptides. Bradykinin sequences are underlined. Species names and the former peptide names are shown in parentheses and square blankets, respectively. In the peptide names, R, EM, and SS mean that the peptides were derived from *G. rugosa*, *G. emeljanove,* and *G. susurra*, respectively.

Twelve cDNA clones were amplified from total RNA from *G. susurra* skin using RT-PCR and 3′-RACE methods. Bioinformatic analyses were performed using the basic local alignment search tool (BLAST) online program (https://blast.ncbi.nlm.nih.gov/Blast.cgi), which revealed that the 12 cDNAs encoded homologs of AMP precursors for four brevinin-2 (preprobrevinin-2SSa, -2SSb, -2SSc, and -2SSd), one esculentin-2 (preproesculentin-2SSa), one ranatuerin-2 (preproranatuerin-2SSa), two acyclic brevenin-1 (preprobrevinin-1SSa and -1SSb), two cyclic brevenin-1 (preprobrevinin-1SSc and -1SSd), one granuliberin (preprogranuliberin-SSa), and one bradykinin (preprobradykinin-SSa). Nucleotide sequences for each set of cDNA are shown in Figure 1. The predicted mature sequence, isoelectric points, and net charges for each set of cDNA are shown in Figure S1. Because of nucleotide sequence similarities between AMP and bradykinin precursors in ranid frogs, cDNAs for bradykinin precursors were sometimes amplified with the same primer sets [17,18]. A comparison of amino sequences of AMPs among *G. susurra*, *G. rugosa*, and *G. emeljanove* is shown in Figure 2. The amino acid sequences of brevinin-2, esculentin-2, brevinin-1, granuliberin, and bradykinin peptides were highly conserved among the three *Glandirana* species. These data supported the reclassification and that *G. rugosa*, *G. emeljanove*, and *G. susurra* are very closely related but not conspecific [15]. BLAST results predicted that brevinin-2SSa, -2SSc, and -2SSd; esculentin-2SSa; ranatuerin-2SSa; and brevinin-1SSc and -1SSd contained a disulfide bond and formed a loop at the C-terminus. The C-termini of brevinin-1SSa and -1SSb and granuliberin-SSa were deduced to be amidated.

Brevinin-2SSb had a highly conserved amino acid sequence with brevinin-2Rb, -2EMa, and -2SSa, but Cys^33^ at the C-terminus commonly found in the other brevinin-2 family peptides had been deleted and replaced with Ser^33^ and an extra seven amino acid residues at the C-terminus had been introduced into the sequence (Figure 2). Ranatuerin-2SSa was the first discovered ranatuerin-2 family peptide in the genus *Glandirana*. Granuliberin was first isolated from the skin of *G. rugosa* with mast cell degranulation activity [19]. Later, its activity against *Staphylococcus aureus* was shown, but such activity against other microbial strains has not yet been reported [20]. Due to the cationic and α-helical properties of their deduced isoelectric points and estimated secondary structure for typical AMPs, in addition to their amino acid sequence novelty, we focused on these three peptides and generated their synthetic replicates. The predicted secondary structures of brevinin-2SSb, ranatuerin-2SSa, and granuliberin-SSa are shown in Figure S2.

Nucleotide sequences of the 12 AMP-precursor cDNA clones have been deposited in the GenBank/EMBL/DDBJ database with the accession numbers LC553543 (brevinin-2SSa), LC553544 (brevinin-2SSb), LC553545 (brevinin-2SSc), LC553546 (brevinin-2SSd), LC553547 (esculentin-2SSa), LC553548 (ranatuerin-2SSa), LC553549 (brevinin-1SSa), LC553550 (brevinin-1SSb), LC553551 (brevinin-1SSc), LC553552 (brevinin-1SSd), LC553553 (granuliberin-SSa), and LC553554 (bradykinin-SSa).

### 2.2. Antimicrobial Activity Assays

**Figure 3:**
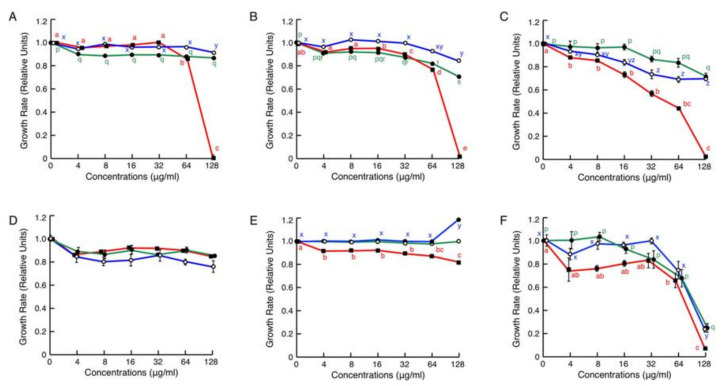
Effects of various concentrations of synthetic brevinin-2SSb (red lines), ranatuerin-2SSa (green lines), and granuliberin-SSa (blue lines) on the growth of animal pathogenic Gram-negative bacteria (**A**–**C**), Gram-positive bacteria (**D**,**E**), and the fungus (**F**). Cells of *Escherichia coli* (**A**), *Salmonella enterica* (**B**), *Pseudomonas aeruginosa* (**C**), *Staphylococcus aureus* (**D**), *Bacillus cereus* (**E**), or *Candida albicans* (**F**) were incubated with serially diluted brevinin-2SSb, ranatuerin-2SSa, or granuliberin-SSa for 20 h at 35 °C. Points and vertical bars represent means and standard error of the mean (SEM), respectively (n = 4). In all panels except (**D**), values with the same letters are not significantly different (*p* ≥ 0.05). The *G. susurra* peptides were not active against the Gram-positive bacterial strains.

In antimicrobial assays against animal pathogenic organisms (Figure 3), brevinin-2SSb was active against the Gram-negative bacteria *Escherichia coli*, *Salmonella enterica*, and *Pseudomonas aeruginosa* and the fungus *Candida albicans*. The growth of these microorganisms was almost completely inhibited by brevinin-2SSb at 128 μg/mL (30 μM), but it was not active or only very slightly active against the Gram-positive bacteria *S. aureus* and *Bacillus cereus*, respectively. Ranatuerin-2SSa and granuliberin-SSa were not active or only slightly active against the bacterial strains examined even at the highest concentration, 128 μg/mL (44 μM and 86 μM, respectively); by contrast, both peptides had significant activity against the fungus *C. albicans*.

**Figure 4:**
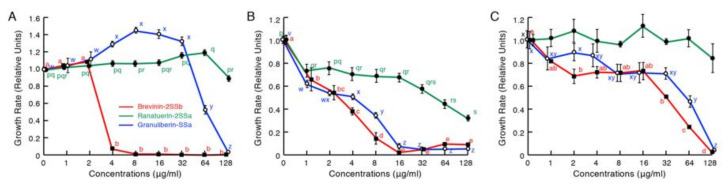
Effects of various concentrations of brevinin-2SSb (red lines), ranatuerin-2SSa (green lines), and granuliberin-SSa (blue lines) on the growth of plant pathogenic Gram-negative bacterium *Xanthomonas oryzae* pv. *oryzae* (**A**), Gram-positive bacterium *Clavibacter michiganensis* subsp. *michiganensis* (**B**), and fungus *Pyricularia oryzae* (**C**). Cells of each bacterial strain or spores of *P. oryzae* were incubated with serially diluted brevinin-2SSb, ranatuerin-2SSa, or granuliberin-SSa for 24 or 96 h at 28 °C, respectively. Points and vertical bars represent means and SEM, respectively (n = 4). In all panels, values with the same letters are not significantly different (*p* ≥ 0.05). Ranatuerin-2SSa was not active against *P. oryzae.*

Subsequently, we examined the antimicrobial activities of these three peptides against plant pathogens (Figure 4). Brevinin-2SSb possessed significant activities and, at concentrations of 4, 16, and 128 μg/mL, completely inhibited the growth of the Gram-negative bacterium *Xanthomonas oryzae* pv. *oryzae*, which causes a serious blight of rice; the Gram-positive bacterium *Clavibacter michiganensis* subsp. *michiganensis*, which causes ring-rot disease in potatoes; and the fungus *Pyricularia oryzae* (synonym *Magnaporthe oryzae*), which causes rice blast disease. Granuliberin-SSa showed activities that were almost equipotent to those of brevinin-2SSb against *C*. *michiganensis* subsp. *michiganensis* and *P. oryzae*. The peptide was also active against *X. oryzae* pv. *oryzae* at concentrations higher than 64 μg/mL. Ranatuerin-2SSa was slightly active against *C. michiganensis* subsp. *michiganensis*; however, no significant effects were detected against *X. oryzae* pv. *oryzae* and *P. oryzae,* even at the highest concentration tested (128 μg/mL).

### 2.3. Morphological Observations

**Figure 5:**
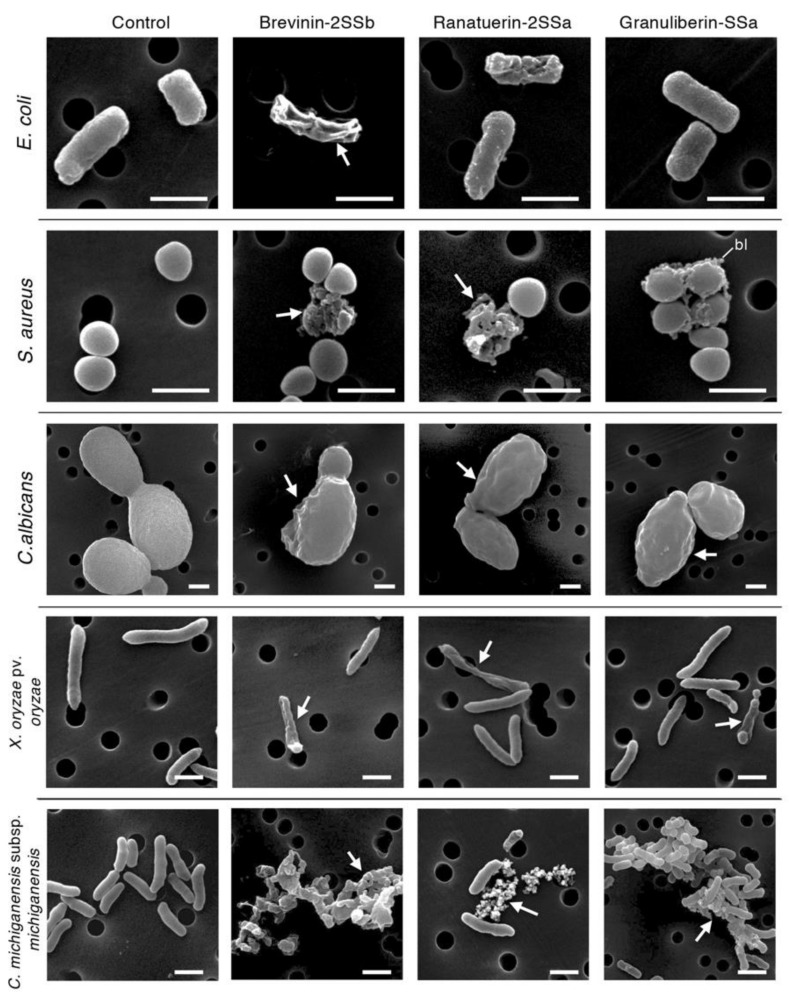
Scanning electron microscopy (SEM) analysis of bacterial and fungal cells following treatment with brevinin-2SSb, ranatuerin-2SSa, or granuliberin-SSa. Aliquots of *E. coli*, *S. aureus*, C*. albicans*, *C. michiganensis* subsp. *michiganensis*, and *X. oryzae* pv. *oryzae* cultures at the midlogarithmic growth phase were incubated with brevinin-2SSb, ranatuerin-2SSa, granuliberin-SSa, or myoglobin (control) at 128 μg/mL for 1 h at room temperature and were then examined using SEM analysis. Abnormally shaped cells indicating cell surface destruction (arrows) or bleb formation (bl) are visible in each panel of brevinin-2SSb-, ranatuerin-2SSa-, and granuliberin-SSa-treated cells when compared with myoglobin-treated cells. Scale bars represent 1 μm.

When evaluating the results of broth microdilution assays, the rate of cell proliferation during incubation periods should be considered, as this sometimes can present a problem in detecting the antimicrobial activities of test substances. Considering this, we investigated the morphology of peptide-treated pathogenic microbe cells using scanning electron microscopy (SEM) analyses. As indicated in Figure 5, control *E. coli*, *S. aureus*, *C. albicans, X. oryzae* pv. *oryzae*, and *C. michiganensis* subsp. *michiganensis* displayed smooth and intact surfaces. Brevinin-2SSa induced cell-surface corrugations on *E. coli*, *C. albicans*, and *X. oryzae* pv. *oryzae* and destroyed *S. aureus* and *C. michiganensis* subsp. *michiganensis* cells. Additionally, the brevinin-2SSb-treated *C. michiganensis* subsp. *michiganensis* cells appeared smaller and more aggregated than the control cells. Similarly, ranatuerin-2SSa induced cell surface corrugations on *E. coli*, C. *albicans,* and *X. oryzae* pv. *oryzae* and diminished the cell membrane integrity of *S. aureus* and *C. michiganensis* subsp. *michiganensis*. Although granuliberin-SSa did not induce any obvious morphological abnormalities on the *E. coli* cells, this peptide induced bleb formation on the surface of *S. aureus* cells. Granuliberin-SSa also induced cell surface corrugations on *C. michiganensis* subsp. *michiganensis* and *X. oryzae* pv. *oryzae* in addition to the destruction and aggregation of *C. michiganensis* subsp. *michiganensis* cells.

### 2.4. Endotoxin Binding Assay

**Figure 6:**
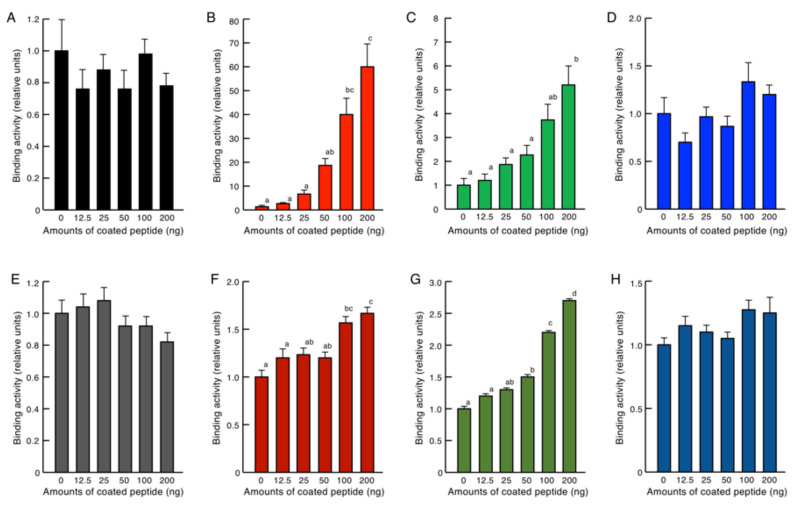
ELEBA evaluation of the abilities of BSA (**A**,**E** for control), brevinin-2SSb (**B**,**F**), ranatuerin-2SSa (**C**,**G**), and granuliberin-SSa (**D**,**H**) to bind to the bacterial endotoxins LPS (**A**–**D**) and LTA (**E**–**H**). Serially diluted brevinin-2SSb, ranatuerin-2SSa, or granuliberin-SSa were coated on 96-well microplate wells and incubated with biotinylated LPS (biotin-LPS) or biotinylated LTA (biotin-LTA) for 4 h at room temperature. Peptide-bound biotin-LPS or biotin-LTA was detected by incubation with HRP-labeled streptavidin followed by incubation with the ELISA POD substrate in the TMB solution, and A_450_ of the reaction products was measured. Columns and vertical bars represent means and SEM, respectively (n = 4). In panels (**B**,**C**,**F**,**H**), values with the same letters are not significantly different (*p* ≥ 0.05). A schematic diagram for the ELEBA is presented in Figure S3. BSA, bovine serum albumin; LPS, lipopolysaccharide; LTA, lipoteichoic acid; ELEBA, enzyme-linked endotoxin binding assay; HRP, horseradish peroxidase; ELISA, enzyme-linked immunosorbent assay; POD, peroxidase; TMB, 3,3’,5,5’-tetramethyl-benzidine.

Cell morphology analysis by SEM strongly suggested that brevinin-2SSb, ranatuerin-2SSa, and granuliberin-SSa associated with bacterial and fungal cell surface substances. To confirm this speculation, we examined the binding abilities of these peptides to lipopolysaccharide (LPS) and lipoteichoic acid (LTA), surface substances of Gram-negative bacteria and Gram-positive bacteria, respectively, using an enzyme-linked endotoxin binding assay (ELEBA) system developed by us. As shown in Figure 6, brevinin-2SSb and ranatuerin-2SSa each bound to LPS and LTA significantly and dose-dependently, although brevinin-2SSb demonstrated higher affinity to LPS and lower affinity to LTA.

### 2.5. Antioxidative Assay

**Figure 7:**
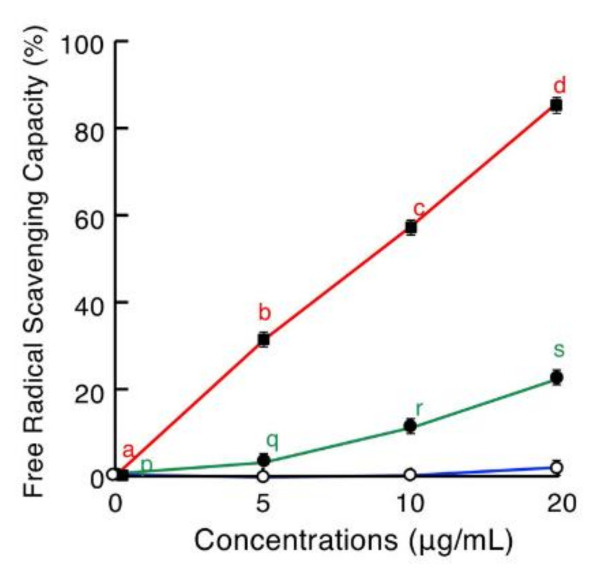
Antioxidative activities of brevinin-2SSb (red line), ranatuerin-2SSa (green line), and granuliberin-SSa (blue line). Serially diluted brevinin-2SSb, ranatuerin-2SSa, or granuliberin-SSa incubated with ABTS^+^ in a U-bottom 96-well microplate at room temperature for 10 min. After incubation, A_734_ values were measured, and the inhibition percentage of ABTS was calculated. Points and vertical bars represent means and SEM, respectively (*n* = 4). Values with the same letters are not significantly different (*p* ≥ 0.05). ABTS, 2,2′-azinobis-(3-ethylbenzothiazoline-6-sulfonate).

Antioxidative activities of brevinin-2SSb, ranatuerin-2SSa, and granuliberin-SSa were evaluated using a standard 2,2′-azinobis-(3-ethylbenzothiazoline-6-sulfonate) (ABTS) method (Figure 7). Brevnin-2SSb possessed strong free-radical scavenging capacity in a dose-dependent manner, and the values at 5, 10, and 20 μg/mL were 29.5%, 55.6%, and 82.3%, respectively. Similarly, albeit more moderately, ranatuerin-2SSa displayed antioxidative activity with free-radical scavenging capacity values at 5, 10, and 20 μg/mL of 2.3%, 8.9%, and 21.9%, respectively. Granuliberin-SSa did not demonstrate any antioxidative activity.

### 2.6. Cytotoxic Assays

**Figure 8:**
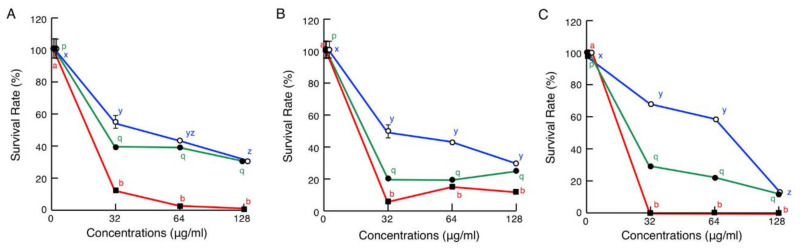
Cytotoxic effects of brevinin-2SSb (red lines), ranatuerin-2SSa (green lines), and granuliberin-SSa (blue lines) in eukaryotic cells. Media containing 5 × 10^3^ COS7 (**A**), HepG2 (**B**), or CPAE (**C**) cells in 500 μL aliquots were incubated with brevinin-2SSb, ranatuerin-2SSa, or granuliberin-SSa at 0, 32, 64, and 128 μg/mL for 24 h at 37 °C, and cell proliferation was determined using standard MTT assay. Cell survival rates were calculated from MTT reduction values and are expressed relative to control (0 dose) cell viability. Points and vertical bars represent means and SEM, respectively (*n* = 4). In all panels, values with the same letters are not significantly different (*p* ≥ 0.05).

Cytotoxicity assays showed that brevinin-2SSb possessed the strongest cytotoxic effects on mammalian cell lines COS7, HepG2, and calf pulmonary artery endothelium (CPAE) among the three peptides tested in the present assay. At a concentration of 32 μg/mL, brevinin-2SSb significantly decreased the survival rates of COS7, HepG2, and CPAE cells to 11.5%, 4.7%, and 0.8%, respectively, and these survival rates remained similar at higher concentrations of brevinin-2SSb (Figure 8). Ranatuerin-2SSa at 32 μg/mL also possessed relatively strong cytotoxic effects on COS7, HepG2, and CPAE cells, yielding survival rates of 38.3%, 19.7%, and 32.9%, respectively, with similar rates at higher concentrations. Granuliberin-SSa demonstrated more moderate cytotoxic effects on COS7 and HepG2 cells than those of brevinin-2SSb and ranatuerin-2SSa, with survival rates of 54.5% and 49.5%, respectively, at 32 μg/mL. In the case of CPAE, granuliberin-SSa significantly decreased the survival rate in a dose-dependent manner. 

**Figure 9:**
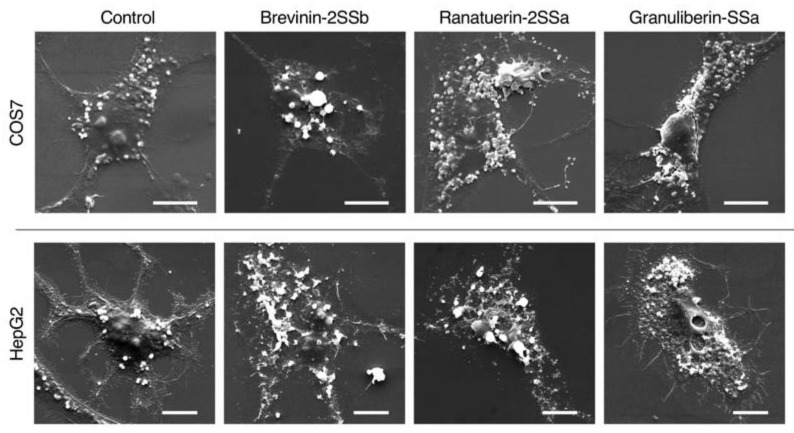
Scanning electron microscopy (SEM) analysis of mammalian cells following treatment with brevinin-2SSb, ranatuerin-2SSa, and granuliberin-SSa. Aliquots of COS7 or HepG2 cells were incubated with brevinin-2SSb, ranatuerin-2SSa, granuliberin-SSa, or myoglobin (control) at 32 μg/mL for 24 h at 37 °C and were then examined using SEM analyses. Cell-membrane destruction is visible in each panel of brevinin-2SSb-, ranatuerin-2SSa-, and granuliberin-SSa-treated cells when compared with control (0 dose) cells. Scale bars represent 10 μm.

We also investigated the effects of brevinin-2SSb, ranatuerin-2SSa, and granuliberin-SSa on the morphology of mammalian cells using SEM analyses. The results demonstrated that all three peptides induced strong cell membrane destruction in COS7 and HepG2 cells (Figure 9).

## 3. Discussion

Wrinkled frogs are distributed in China, North and South Korea, and Japan. Classically, the wrinkled frogs in Japan and Korea were classified as the same species and named *Rana rugosa*, but they have now been reclassified as different species in the genus *Glandirana* and named *G. rugosa* and *G. emeljanove*, respectively [21]. Earlier studies of structures and biological activities of “*R. rugosa*” skin AMPs and/or their genes have been reported [22,23,24,25]. Previously, *R. rugosa* AMPs were divided into two groups, namely rugosins and gaegrins; the former ones were found in the Japanese species (G*. rugosa*), and the latter were found in the Korean species (*G. emeljanove*). However, based on amino acid sequence comparisons, Won et al. [26] reclassified rugosins and gaegrins into known frog AMP families such as brevinin-1, brevinin-2, and esculentin-2. In the present study, we adopt this rule to classify our cDNA clones. Previous reports have demonstrated that brevinin-2Ra (rugosin A), brevinin-2Rb (rugosin B), esculentin-2R (rugosin C), brevinin-2EMa (gaegrin-1), brevinin-2EMb (gaegrin-2), brevinin-2EMb′ (gaegrin-3), esculentin-2EM (gaegrin-4), brevinin-1EMa (gaegrin-5), and brevinin-1EMb (gaegrin-6) exhibit antimicrobial activities [22,23,24].

The brevinin-2 family peptides were first isolated from the skin of the Japanese pond frog *Rana brevipoda porosa*, now reclassified as *Pelophylax porosus* [27]. Based on amino acid sequence similarities, brevinin-2 family peptides in the Japanese and Korean wrinkled frogs can be divided into two groups: (1) the brevinin-2R (rugosin A) group, consisting of brevinin-2EMb (gaegrin-2), -2EMb′ (gaegrin-3), -2SSd, and -2SSe, and (2) the brevinin-2Rb (rugosin B) group, consisting of brevinin-2EMa, -2SSb, and -2SSc. The primary structures are poorly conserved between brevinin-2 peptides belonging to the rugosin A group and rugosin B group, and their sequence similarities are approximately 30%–35%. Both types of brevinin-2 family peptides are commonly found in the three *Glandirana* species. Although the primary structures of the brevinin-2 family peptides are poorly conserved among species, most show conservation of a *Rana* box at the C-terminus, which generates an intrachain disulfide bond [28]. However, brevinin-2SSb lacks this motif and is characterized by different structural features and a predicted secondary conformation that can be distinguished from that of brevinin-2Rb; this is particularly the case with respect to the extent of α-helical and β-sheet conformations that mediate both the antimicrobial activity and the hemolytic cytotoxicity of these peptides [29].

Ranatuerin-2 family peptides were first identified in the skin of the bullfrog *Lithobates catesbeianus* [30]. The family has been found in North American and Eurasian frog species. The primary structures of the ranatuerin-2 peptides have been poorly conserved, and antimicrobial activities of members of the family are highly variable [31]. Ranatuerin-2SSa was the first identified ranatuerin-2 family peptide in the genus *Glandirana*. According to BLAST search results, ranatuerin-2SEa found in *Rana sevosa* (now reclassified as *Lithobates sevosus*) had the highest amino acid sequence similarity to ranatuerin-2SSa, but the value was only 62%. According to the predicted secondary structures, the α-helical and β-sheet contents of ranatuerin-2SSa were lower and higher, respectively, than those identified in ranatuerin-2 from *L. catesbeianus*. These results suggest that ranatuerin-2SSa may have reduced antimicrobial activity and increased toxicity when compared to ranatuerin-2 [29].

Brevinin-1 family peptides were also first isolated from *P. porosus* [27]. This peptide family can be divided into two types: cyclic brevinin-1 and acyclic brevinin-1 [32]. Cyclic brevinin-1 possesses a disulfide bond in the C-terminal region; however, acyclic brevinin-1 lacks Cys residues, which prevent the formation of disulfide bonds, but its C-terminus is amidated. While cyclic brevinin-1 compounds were found in *G. emeljanove* and *G. susurra,* acyclic brevinin-1 was only found in *G. susurra.*

Granuliberin family peptides have been identified in *G. rugosa* and *G. susurra*. Interestingly, the deduced amino acid sequence following the stop codon in the granuliberin precursor was very close to that of the N-terminal seven-amino-acid residues of brevinin-1SSc and -1SSd, suggesting that granuliberin and brevinin-1 may be derived from a common ancestor gene. The secondary structure predictions suggested that these peptides exist in β-sheet and coiled-coil conformation; this conformation predicts relatively low antimicrobial activity and high toxicity [29].

In the present study, we generated brevinin-2SSb, ranatuerin-2SSa, and granuliberin-SSa, focusing on their sequence novelty and biochemical features generally found in typical AMPs. In the microdilution assays, these three peptides significantly inhibited the growth of *C. albicans*. *Candida* species induce cutaneous and systemic infections, especially in immunocompromised patients. A high mortality rate in these patients in addition to the emergence of resistance to the most common antifungal drugs has necessitated the development of alternative antifungal agents [33]. The three novel peptides studied herein can be regarded as potential candidates for antifungal agents. Moreover, although ranatuerin-2SSa and granuliberin-2SSa did not show remarkable antibacterial activities in the broth microdilution assays, these peptides and brevinin-2SSb induced morphological abnormalities on cells of the examined bacterial strains. 

An increase in antibiotic-resistant plant pathogenic microorganisms is also becoming a severe problem in agriculture. For example, the resistance to streptomycin, a traditional antibiotic for bacterial disease in plants as well as animals, has now been characterized in a wide range of plant pathogens on a global scale [34]. Similarly, kasugamycin- and oxolinic-resistant plant pathogens are also increasing [34]. Thus, development of new antimicrobial resources is urgently needed. Previously, Shi et al. [35] reported that anti-*X. oryzae* pv. *oryzae* activity of melittin occurs through the destruction of the cell membrane integrity. In this study, ranatuerin-2SSa and granuliberin-SSa displayed remarkable antimicrobial activity against Gram-negative *X. oryzae* pv. *oryzae* and Gram-positive *C. michiganensis* subsp. *michiganensis* plant pathogens in broth microdilution assays. SEM observations demonstrated that the three *G. susurra* peptides induced obvious morphological abnormalities on *X. oryzae* pv. *oryzae* cells similar to those of cells treated with melittin. Moreover, ranatuerin-2SSa and granuliberin-SSa inhibited the growth of the fungal cells of *P. oryzae.* These results suggested that frog AMPs may contribute to the development of a new agent for antibiotic-resistant plant pathogens. 

The first barrier against antimicrobials in a microorganism is the microbial cell wall [36]. In the case of Gram-negative bacteria, the outer membrane is the outermost barrier and contains LPS located in the outer leaflet, whereas Gram-positive bacteria lack the outer membrane but have a thicker peptidoglycan layer [37]. LTA is also an important cell-surface component in Gram-positive bacteria. In this study, we attempted to detect the interaction between *G. susurra* AMPs and LPS or LTA. The chromogenic *Limulus amoebocyte* lysate (LAL) assay is a widely accepted sensitive system for the detection of trace amounts of LPS. This system can be applied to detect the LPS-binding ability of peptides and proteins [38]. We previously investigated the LPS-binding ability of synthetic chicken cathelicidin-B1 peptide using this system and demonstrated that it requires peptide concentrations higher than 12.5 μg/mL [39]. In a pilot experiment in this study, we observed that ELEBA could detect the LPS-binding ability of the peptide at 200 ng/mL (data not shown). When protein concentrations in the standard curves between LAL and ELEBA systems are compared, the detection sensitivity of ELEBA is estimated to be approximately 50 to 100 times greater than that of the LAL assay system. The electrophoretic mobility shift assay is an established method to evaluate the LTA-binding ability of proteins [39,40,41], but it is semiquantitative. We previously detected the LTA-binding ability of histone H3 at a concentration of 200 μg/mL using the mobility shift assay [39], and in a pilot experiment in this study, we observed the LTA-binding of the same protein at a concentration of 400 ng/mL by ELEBA (data not shown). Thus, ELEBA is a useful quantitative system for the detection of both Gram-negative and Gram-positive bacterial endotoxins. Herein, we showed that brevinin-2SSb and ranatuerin-2SSa bound to LPS and LTA, demonstrating that these two peptides associate with *E. coli* and *S. aureus* cells through the binding of cell-surface LPS and LTA, respectively. Dong et al. [42] reported that chensinin-1, a natural AMP isolated from the skin of Chinese brown frog *Rana chensinensis,* mitigated the effects of LPS and decreased LPS-inducible production of proinflammatory cytokines tumor necrosis factor-α and interleukin-6 in RAW264.7 cells. Thus, ranatuerin-2SSa and brevinin-2SSb are expected to neutralize the endotoxins and act as anti-inflammatory agents like chensinin-1. 

In addition to the defensive function against invasion by bacteria and fungi, amphibian skin plays an important role in protecting the organism from external harmful factors such as ultraviolet (UV) radiation. UV exposure has been linked to the generation of reactive oxygen species [43]. Thus, some skin peptides in amphibians possess antioxidant activity [44,45,46]. In this study, brevinin-2SSb and ranatuerin-2SSa possessed strong and weak antioxidative activity, respectively, via a conventional ABTS assay. Such activity was attributed to the presence of Met, Tyr, Pro, Cys, and Trp. In particular, the sulfhydryl group in Cys residues and the indole ring in Trp possess higher activities in free-radical scavenging [44]. In the amino acid sequences, brevinin-2SSb contains more of the above five amino acids than the other two peptides, perhaps contributing to the higher antioxidant activity of brevinin-2SSb. Although two Cys residues were present in the ranatuerin-2SSa sequence, their SH groups were donated for disulfide bond formation. 

The present data show that brevinin-2SSb, ranatuerin-2SSa, and granuliberin-SSa have potential as natural antibiotics, particularly for agriculture or horticulture. While brevinin-2SSb, ranatuerin-2SSa, and granuliberin-SSa may present several advantages, these peptides elicited comparatively high levels of cytotoxicity at their effective antibacterial concentrations. These results suggest that structural alterations, including amino acid substitutions, relocations, and/or incorporation of D-amino acids, might be introduced in an effort to reduce cytotoxicity [29,47,48,49,50].

## 4. Materials and Methods

All experiments were approved by the Toho University Biosafety Committee for Recombinant DNA Experiments (18-53-327), Animal Care and User (17-41-345), and Pathogens (18-51-103), and were performed by authorized investigators.

### 4.1. Bacterial and Fungal Cell Strains

Animal pathogenic bacterial cell strains of the Gram-negative *E. coli* (JCM5491), *S. enterica* (JCM1652), and *P. aeruginosa* (JCM6119) and the Gram-positive *S. aureus* (JCM2874) and *B. cereus* (JCM2152), and a fungal strain of *C. albicans* (JCM2085), were purchased from the Japan Collection of Microorganisms (Riken Bioresource Center, Tsukuba, Japan). The Gram-negative and Gram-positive plant pathogenic bacteria, *X. oryzae* pv. *oryzae* (MAFF311018) and *C. michiganensis* subsp. *michiganensis* (MAFF301254), respectively, and a plant pathogenic fungus, *P. oryzae* (MAFF101511), were purchased from Genetic Resources Center (National Agriculture and Food Research Organization, Tsukuba, Japan). The cell strains were grown on appropriate agar plates and then inoculated in the growth medium recommended by the suppliers for secondary culture for antimicrobial assays.

### 4.2. Mammalian Cell Lines

African green monkey kidney derived COS7 cells (an immortal cell line), human liver hepatocellular carcinoma derived HepG2 cells (a tumor cell line), and calf pulmonary artery endothelium (CPAE) cells (a normal cell line), were purchased from Health Science Research Resource Bank (Osaka, Japan). COS7 and HepG2 cells and CPAE cells were cultured in Dulbecco’s modified Eagle’s medium (DMEM; Nissui, Tokyo) and minimum essential medium (MEM), respectively, each supplemented with 10% fetal bovine serum (FBS; Sigma-Aldrich, St. Louis, MO, USA) and antibiotics (100 U/mL penicillin, 100 μg/mL streptomycin; Life Technologies, Carlsbad, CA, USA). All cell lines were cultured at 37 °C under 5% CO_2_–95% air conditions.

### 4.3. Synthetic Peptides

Putative *G. susurra* brevinin-2SSb (SLFSLIKAGA^10^ KFLGKNMLKQ^20^ GPQYPACKVS^30^ KDSENVNWKS^40^), ranatuerin-2SSa (GLISTIWNTA^10^ SNVAGTLTDS^20^ VKCKFKKC), and granuliberin-SSa (FIFLPIFRRP^10^ VS.NH_2_) peptides in predicted mature forms (Figure S1) were obtained from the Biologica Company (Nagoya, Japan) at purities of 97%, 85%, and 92%, respectively. A disulfide bond was introduced between Cys^23^ and Cys^28^ in ranatuerin-2SSa. The synthetic peptides were diluted to 5 mg/mL as stock solutions in dimethyl sulfoxide and were stored at −20 °C.

### 4.4. Amplification of AMP Precursor cDNAs

Four adult *G. susurra* specimens (39–55 mm in body length) were collected from a paddy field on Sado Island by authorized investigators. Frogs were anesthetized by immersion in ice-cold water and sacrificed by decapitation. Skins were immediately removed and pooled, and total RNA was extracted using a modified acid phenol/guanidine isothiocyanate procedure [51]. The open reading frames of AMP precursor cDNAs were amplified using RT-PCR in volumes of 50 μL using a One-Step RT-PCR Kit (Qiagen, Chatsworth, CA, USA). Total RNA (100 ng) samples were incubated with sets of gene-specific reverse primers and a common forward primer at 50 °C for 30 min for reverse-transcription and then at 95 °C for 15 min for denaturation of reverse transcriptase. Subsequently, PCR was performed under the following conditions: 5 min at 94 °C for DNA denaturation followed by 35 cycles of 30 s at 94 °C, 30 s at 55 °C, and 1 min at 72 °C, with a final extension step of 7 min at 72 °C. The forward primer 5′-ATGTTCACCATGAAGAAATC-3′ was designed according to the nucleotide sequence of a highly conserved region of the signal peptide region of ranid frog AMP precursors [12,17]. Four reverse primers, 5′-ATCAGACGTTCAGCCAAATGA-3′, 5′-AGATGATTTCCAATTCCAT-3′, 5′-CTATCCCACATCAGGAGACTTTCC-3′, and 5′-AGACATCTGTTGTGCCCTTTA-3′, were examined according to our previous reports [12,13,17,18]. AMP precursor cDNAs were also amplified from 200 ng of the skin total RNA specimens using a 3′-Full RACE Core Set (Takara, Ohtsu, Japan) according to the manufacturer’s protocol. In brief, the 3′-RACE reactions were performed on a 20 μL reaction scale using an Oligo dT-3sites Adaptor Primer Mix and Avian myeloblastosis virus (AMV) reverse transcriptase at 30 °C for 10 min, 50 °C for 30 min, 95 °C for 5 min, and 5 °C for 5 min. The reaction was incubated with the above-mentioned forward primer, the 3-site adaptor primer in the set, dNTP mixture, and Ex Taq DNA polymerase on a 100 μL reaction scale. Oligonucleotides for PCR primers were provided by Sigma Genosys (Ishikari, Japan). PCR products were separated by electrophoresis on 1.5% agarose gels, stained with ethidium bromide, and then visualized using a UV transilluminator. Amplified DNAs of appropriate sizes were excised and purified from gels and then subcloned into pSTBlue-1 vector using the AccepTor Vector Kit (Novagen, Darmstadt, Germany). Nucleotide sequence analyses were performed using the dideoxy chain termination method with a Big-Dye Terminator Cycle Sequencing Kit (Applied Biosystems, Foster City, CA, USA) by Eurofins Genomics Company (Tokyo). Nucleotide and amino acid sequence identities and predicted isoelectric points and secondary structures were analyzed using Genetyx-Mac version 15.0.1 software (Software Development Corporation, Osaka, Japan).

### 4.5. Antimicrobial Assays

Antimicrobial activities of serially diluted synthetic peptides against animal pathogenic organisms were determined in 100 μL of cation-adjusted Mueller–Hinton broth (CAMHB) (Becton and Dickinson, Franklin Lakes, NJ, USA) inoculated with log phase cultures (10 μL of 5 × 10^5^ colony forming units/mL) of the cells of *E. coli*, *E. aerogenes*, *S. enterica, P. aeruginosa*, *B. cereus,* and *C. albicans* in 1% BSA-coated 96-well microtiter cell culture plates at 35 °C in normal air. After incubation for 20 h, absorbance at 595 nm (A_595_) of each well was measured using an iMark microplate absorbance reader (Bio-Rad, Hercules, CA, USA). Similarly, inocula of *X. oryzae* pv. *oryzae* and *C. michiganensis* subsp. *michiganensis* were incubated in CAMHB and LB broth, respectively, at 28 °C for 24 h. For the anti-plant-fungus assay, spores of *P. oryzae* grown on a potato dextrose agar plate at 28 °C were suspended in potato dextrose broth (PDB) after removal of hyphae and then filtered by puluriStrainer Mini 20 (Funakoshi, Tokyo). The spore suspension obtained by the filtration was diluted to 2 × 10^4^ cells/mL with PDB, and 80-μL aliquots were incubated with the peptides in 96-well plates at 28 °C for 96 h. In each assay, the final concentrations of peptides ranged from 0 to 128 μg/mL.

### 4.6. Scanning Electron Microscopy (SEM)

For antimicrobial experiments, bacterial and fungal cells were grown to A_595_ ≈ 0.6 in 1.5 mL tubes containing 400 μL of LB broth and incubated with brevinin-2SSa, ranatuerin-2SSa, granuliberin-SSa, or myoglobin (control) at 128 μg/mL of *E. coli*, *S. aureus*, *C. albicans*, *X. oryzae* pv. *oryzae,* and *C. michiganensis* subsp. *michiganensis* for 1 h at room temperature. Cells were harvested by gentle centrifugation, prefixed with 2.5% glutaraldehyde for 1 h, fixed in 1% OsO_4_ for 1 h, and washed with phosphate buffer. Cells were dehydrated in an ethanol series (50%, 70%, 90%, 95%, and 100%) on a nano-percolator filter (JEOL, Tokyo, Japan), incubated with ethanol/t-butyl alcohol (1:1) for 1 h, and incubated with 100% t-butyl alcohol three times for 20 min each. Samples were then frozen at 4 °C, lyophilized, and coated with gold particles (15 nm) using a Quick Coater SC701 (Sanyu Electron, Tokyo, Japan). Morphological observations were then performed using a JSM-6390LV SEM instrument (JEOL). For cytotoxic experiments, COS7 and HepG2 cells were precultured on poly-l-lysine coated 12 mm cover slips (Corning) placed in each well of a 24-well cell culture plate, followed by incubation with the *G. susurra* AMPs under the same experimental conditions as described for the cytotoxic assay. After incubation, SEM samples were prepared as described above and subjected to SEM analysis.

### 4.7. Enzyme-Linked Endotoxin Binding Assay (ELEBA)

To assess binding abilities of brevinin-2SSa, rantuerin-2SSa, and granuliberin-SSa to bacterial endotoxins, such as LPS and LTA, we developed a standard ELISA-based assay method, ELEBA (Figure S3). Each well of a 96-well Nunc Immobilizer Amino microtiter plate (Thermo Scientific) was filled with 100 μL of serially diluted brevinin-2SSa, ranatuerin-2SSa, or granuliberin-SSa (0–2 mg/mL in 100 mM carbonate buffer, pH 9.6) and incubated overnight at 4 °C with gentle agitation for coupling. After incubation, the wells were aspirated, and 300 μL of 10 mM ethanolamine in carbonate buffer was added. Wells were then incubated for 1 h at 4 °C in the dark for postcoupling. The wells were aspirated and washed with 300 μL of phosphate buffered saline (PBS; pH7.2) containing 0.05% (*w*/*v*) Tween 20 (PBS-T) three times. To each well, 300 μL of the blocking reagent for ELISA (Sigma-Aldrich) was added, and wells were incubated for 1 h at room temperature. The wells were aspirated, washed with PBS-T, and incubated with biotinylated LPS (biotin-LPS) from *E. coli* O111:B4 in 100 μL of the blocking reagent for 2 h at room temperature. The biotin-LPS was purchased from InvivoGen (San Diego, CA, USA), dissolved in H_2_O (500 μg/mL), and then diluted to 1:1000 (*v*/*v*) in the blocking reagent. To quantify LTA binding, biotinylated *S. aureus* LTA (biotin-LTA) prepared according to Baik et al. [52] was used. LTA (1 mg; InvivoGen) was conjugated with 500 μg of biotin-2-sulfo-*N*-hydroxysuccinimide ester sodium salt (biotin-NHS; Sigma Genosys) at room temperature for 4 h with gentle agitation. After conjugation, unbound biotin-NHS was separated and discarded using a Microcon YM3 centrifugal filter unit (Merck-Millipore; Burlington, MA, USA). The final volume of the biotin-LTA sample solution was adjusted to 500 μL with PBS and then diluted to 1:32,000 (*v*/*v*) in the blocking reagent. After incubation with biotin-LPS or biotin-LTA, the wells were aspirated, washed with PBS-T, and incubated with 100 μL of streptavidin-conjugated horseradish peroxidase (SA-HRP; PerkinElmer, Waltham, MA, USA) diluted in the blocking reagent for 30 min at room temperature. The wells were aspirated and then washed with PBS-T and PBS, before being reacted with 100 μL of the substrate solution in ELISA POD Substrate TMB kit (Nakalai Tesque, Kyoto, Japan) for 15 min at room temperature in the dark. The reaction was stopped by the addition of 100 μL of 1 M H_2_SO_4_. Finally, the A_450_ of the specimens was measured using a microtiter plate reader.

### 4.8. Antioxidative Assay

Antioxidative activity was determined by 2,2*′*-azinobis-(3-ethylbenzothiazoline-6-sulfonate) (ABTS) cation radical scavenging assay as described previously [53]. Briefly, to produce ABTS radical cation (ABTS^+^), 2.5 mL of 14 mM ABTS (Sigma Aldrich) and 88 μL of 140 mM potassium persulfate were mixed to stand in the dark at room temperature for 16 h (stock solution) and then diluted to 1:88 with PBS just before use (working solution). The working solution (180 μL aliquot) was incubated with 20 μL of brevinin-2SSa, ranatuerin-2SSa, or granuliberin-SSa solutions at final concentrations of 0, 5, 10, and 20 μg/mL in the wells of a U-bottom 96-well microplate at room temperature for 10 min; then, A_734_ values of the specimens were measured. The inhibition percentage of ABTS was calculated using following formula:

ABTS cation radical scavenging activity (%) = [(A _blank_ − A _sample_)/A _blank_] × 100

### 4.9. Cytotoxicity Assays

To assess cytotoxic the effects of the *G. susurra* AMPs on eukaryotic cells, standard MTT assays were performed according to our previous study [13]. Briefly, 5 × 10^3^ COS7, HepG2, or CPAE cells were cultured in wells of collagen-coated 96-well microtiter cell culture plates (Thermo Fisher Scientific, Waltham, MA, USA) containing 100 μL of DMEM supplemented with 10% FBS and antibiotics at 37 °C overnight in an atmosphere of 5% CO_2_. Media were then replaced with fresh media containing brevinin-2SSa, ranatuerin-2SSa, or granuliberin-SSa at final concentrations of 0, 32, 64, and 128 μg/mL, and cells were incubated for 24 h. Media were then replaced with 100 μL of fresh media containing 0.5% 3-(4,5-dimethylthiazol-2-yl)-2,5-diphenyltetrazolium bromide (MTT) (Wako, Osaka), and cells were incubated for 4 h in the dark. Aliquots (100 μL) of lysis buffer containing 6 N HCl/isopropanol (0.34/99.66, *v*/*v*) were added and incubated overnight under the same conditions. Finally, A_570_ values of specimens were measured using a microtiter plate reader. Survival rates were expressed according to MTT reduction values, which were calculated relative to the survival rates of control (0 dose for 24 h incubation) cells.

### 4.10. Statistical Analyses

Statistical analyses of data from antimicrobial, bacteria-killing kinetics, and cytotoxic assays were performed using analysis of variance (ANOVA) followed by multiple comparisons using Scheffé’s F test. Differences were considered significant when *p* < 0.05.

## 5. Conclusions

We cloned cDNAs encoding biosynthetic precursors for brevinin-2, esculentin-2, ranatuerin-2, cyclic and acyclic brevinin-1, granuliberin, and bradykinin family peptides from the skin of *G. susurra* frogs, which were recently classified as a new *Glandirana* species. Among these peptides, we focused on three novel peptides, brevinin-2SSb, ranatuerin-2SSa, and granuliberin-SSa, and examined their antimicrobial activities against animal and plant pathogens. Brevinin-2SSb displayed antimicrobial activity against not only animal pathogenic strains of *E. coli*, *S. enterica*, *P. aeruginosa*, and *C. albicans,* but also plant pathogenic strains of *X. oryzae* pv. *oryzae*, *C. michiganensis* subsp. *michiganensis*, and *P. oryzae*. Ranatuerin-2SSa and granuliberin-SSa exhibited antimicrobial properties against plant pathogens. These peptides also induced morphological abnormalities on the microbes examined in this study. The ELEBA system developed to evaluate the endotoxin-biding abilities of peptides revealed that brevinin-2SSb and ranatuerin-2SSa both possessed strong binding affinity to LPS and moderate binding affinity to LTA. We also observed the cytotoxic activity of these peptides, warranting their further development as therapeutic agents, albeit with modifications that reduce toxicity.

## Supplementary Materials

The following are available online at https://www.mdpi.com/2079-6382/9/8/457/s1, Figure S1: Deduced amino acid sequences of the mature *G. susurra* peptides, Figure S2: Predicted secondary structures of brevinin-2SSb, ranatuerin-2SSa, and granuliberin-SSa, Figure S3: A schematic diagram of ELEBA.

## References

[1] Cheesman M.J., Ilanko A., Blonk B., Cock I.E. (2017). Developing new antimicrobial therapies: Are synergistic combinations of plant extracts/compounds with conventional antibiotics the solution?. Pharmacogn. Rev..

[2] Auvynet C., Rosenstein Y. (2009). Multifunctional host defense peptides: Antimicrobial peptides, the small yet big players in innate and adaptive immunity. FEBS J..

[3] Zasloff M. (2002). Antimicrobial peptides of multicellular organisms. Nature.

[4] Pushpantathan M., Gunasekaran P., Rajendhran J. (2013). Antimicrobial peptides: Versatile biological properties. Int. J. Pept..

[5] Tossi A., Sandri L., Giangaspero A. (2000). Amphipathic, α-helical antimicrobial peptides. Biopolymers.

[6] Lei J., Sun L., Huang S., Zhu C., Li P., He J., Mackey V., Coy D., He Q. (2019). The antimicrobial peptides and their potential clinical applications. Am. J. Transl. Res..

[7] Dobson A.J., Purves J., Rolff J. (2014). Increased survival of experimentally evolved antimicrobial peptide-resistant *Staphylococcus aureus* in an animal host. Evol. App..

[8] Conlon J.M. (2008). Reflections on a systematic nomenclature for antimicrobial peptides from the skins of frogs of the family Ranidae. Peptides.

[9] Ladram A., Nicolas P. (2016). Antimicrobial peptides from frog skin: Biodiversity and therapeutic promises. Front. Biosci..

[10] Gissi C., San Mauro D., Pesole G., Zardoya R. (2006). Mitochondrial phylogeny of Anura (*Amphibia*): A case study of congruent phylogenetic reconstruction using amino acid and nucleotide characters. Gene.

[11] Nicolas P., Vanhoye D., Amiche M. (2003). Molecular strategies in biological evolution of antimicrobial peptides. Peptides.

[12] Ohnuma A., Conlon J.M., Iwamuro S. (2010). Differential expression of genes encoding preprobrevinin-2, prepropalustrin-2, and preproranatuerin-2 in developing larvae and adult tissues of the mountain brown frog *Rana ornativentris*. Comp. Biochem. Phys. C Toxicol Pharmacol..

[13] Ogawa D., Mochitate M., Furukawa M., Hasunuma I., Kobayashi T., Kikuyama S., Iwamuro S. (2017). Molecular cloning and functional characterization of antimicrobial peptides brevinin-1ULf and ulmin-1ULa in the skin of the newly classified Ryukyu brown frog *Rana ulma*. Zool. Sci..

[14] Sekiya K., Ohtani H., Ogata M., Miura I. (2010). Phyletic diversity in the frog *Rana rugosa* (*Anura: Ranidae*) with special reference to a unique morphotype gound from Sado Island, Japan. Curr. Herpetol..

[15] Sekiya K., Miura I., Ogata M. (2012). A new frog species of the genus *Rugosa* from Sado Island, Japan (*Anura, Ranidae*). Zootaxa.

[16] Shioda T. (2015). A Comparison of iris color pattern between *Glandirana susurra* and *G. rugosa* (*Amphibia, Anura*, *Ranidae*). Curr. Hepetol..

[17] Suzuki H., Iwamuro S., Ohnuma A., Coquet L., Leprince J., Jouenne T., Vaudry H., Taylor C.K., Abel P.W., Conlon J.M. (2007). Expression of genes encoding antimicrobial and bradykinin-related peptides in skin of the stream brown frog *Rana sakuraii*. Peptides.

[18] Tazato S., Conlon J.M., Iwamuro S. (2010). Cloning and expression of genes encoding antimicrobial peptides and bradykinin from the skin and brain of Oki Tago’s brown frog, *Rana tagoi okiensis*. Peptides.

[19] Nakajima T., Yasuhara T. (1977). A new mast cell degranulating peptide, granuliberin-R, in the frog (*Rana rugosa*) skin. Chem. Pharm. Bull..

[20] Nakao S., Komagoe K., Inoue T., Katsu T. (2011). Comparative study of the membrane-permeabilizing activities of mastoparans and related histamine-releasing agents in bacteria, erythrocytes, and mast cells. Biochim. Biophys. Acta.

[21] Frost D.R. (2017). Amphibian Species of the World 6.0, an Online Reference. Electronic Database Accessible at American Museum of Natural History, New York. https://amphibiansoftheworld.amnh.org/index.php.

[22] Park J.M., Jung J.E., Lee B.J. (1994). Antimicrobial peptides form the skin of a Korean frog, *Rana rugosa*. Biochem. Biophys. Res. Comuun..

[23] Park J.M., Jung J.E., Moon H.M., Lee B.J. (1995). Molecular cloning of cDNAs encoding precursors of frog skin antimicrobial peptides from *Rana rugosa*. Biochim. Biophys. Acta.

[24] Suzuki S., Ohe Y., Okubo T., Kakegawa T., Tatemoto K. (1995). Isolation and characterization of novel antimicrobial peptides, rugosins A, B and C, from the skin of the frog, *Rana rugosa*. Biochem. Biophys. Res. Commun..

[25] Kwon S.Y., Carlson B.A., Park J.M., Lee B.J. (2000). Structural organization and expression of the gaegurin 4 gene of *Rana rugosa*. Biochim. Biophys. Acta.

[26] Won H.S., Kang S.J., Lee B.J. (2009). Action mechanism and structural requirements of the antimicrobial peptides, gaegurins. Biochim. Biophys. Acta.

[27] Morikawa N., Hagiwara K., Nakajima T. (1992). Brevinin-1 and -2, unique antimicrobial peptides from the skin of the frog, *Rana brevipoda porsa*. Biochem. Biophys. Res. Comuun..

[28] Clark D.P., Durell S., Maloy W.L., Zasloff M. (1994). Ranalexin. A novel antimicrobial peptide from bullfrog (*Rana catesbeiana)* skin, structurally related to the bacterial antibiotic, polymyxin. J. Biol. Chem..

[29] Mai X.T., Huang J., Tan J., Huang Y., Chen Y. (2015). Effects and mechanisms of the secondary structure on the antimicrobial activity and specificity of antimicrobial peptides. J. Pep. Sci..

[30] Goraya J., Knoop F.C., Conlon J.M. (1998). Ranatuerins: Antimicrobial peptides isolated from the skin of the American bullfrog, *Rana catesbeiana*. Biochem. Biophys. Res. Comuun..

[31] Conlon J.M., Kolodziejek J., Nowotny N. (2009). Antimicrobial peptides from the skins of North American frogs. Biochim. Biophys. Acta.

[32] Conlon J.M., Sonnevend A., Jouenne T., Coquet L., Cosquer D., Vaudry H., Iwamuro S. (2005). A family of acyclic brevinin-1 peptides from the skin of the Ryukyu brown frog *Rana okinavana*. Peptides.

[33] Roscetto E., Contursi P., Vollaro A., Fusco S., Notomista E., Catatani M.S. (2018). Antifungal and anti-biofilm activity of the first cryptic antimicrobial peptide from an archaeal protein against *Candida* spp. Clinical isolates. Sci. Rep..

[34] Sundin G.W., Wang N. (2018). Antibiotic resistance in plant-pathogenic bacteria. Annu. Rev. Phytopathol..

[35] Shi W., Li C., Zong X., Han D., Chen Y. (2016). Antimicrobial peptide melittin against *Xanthomonas oryzae* pv. *oryzae*, the bacterial leaf blight pathogen in rice. Appl. Microbiol. Biotechnol..

[36] Martínez de Tejada G., Sánchez-Gómez S., Rázquin-Olazaran I., Kowalski I., Kacoins Y., Heinbockel L., Andrä J., Schürholz T., Hornef M., Dupont A. (2012). Bacterial cell wall compounds as promising targets of antimicrobial agents I. Antimicrobial peptides and lipopolyamines. Curr. Drug Targets.

[37] Epand R.M., Walker C., Epand R.F., Magarvey N.A. (2016). Molecular mechanisms of membrane targeting antibiotics. Biochim. Biophys. Acta.

[38] Tack B.F., Sawai M.V., Kearney W.R., Robertson A.D., Sherman M.A., Wang W., Hong T., Boo L.M., Wu H., Waring A.J. (2002). SMAP-29 has two LPS-binding sites and a central hinge. Eur. J. Biochem..

[39] Takeda A., Tsubaki T., Sagae N., Onda Y., Inada Y., Mochizuki T., Okumura K., Kikuyama S., Kobayashi T., Iwamuro S. (2014). Bacterial toxin-inducible gene expression of cathelicidin-B1 in the chicken bursal lymphoma-derived cell line DT40: Functional characterization of cathelicidin-B1. Peptides.

[40] Stinson M.W., Mcaughlin R., Choi S.H., Juarez Z.E., Barnard J. (1998). *Streptococcal* histone-like protein: Primary structure of hlpA and protein binding to lipoteichoic acid and epithelial cells. Infect. Immun..

[41] Morita S., Tagai C., Shiraishi T., Miyaji K., Iwamuro S. (2013). Differential mode of antimicrobial actions of arginine-rich and lysine-rich histones against Gram-positive *Staphylococcus aureus*. Peptides.

[42] Dong W., Sun Y., Shang D. (2015). Interactions between chensinin-1, a natural antimicrobial peptide derived from *Rana chensinensis*, and lipopolysaccharide. Biopolymers.

[43] Barbosa E.A., Oliveira A., Placido A., Socodato R., Portugal C.C., Leite J. (2018). Structure and function of a novel antioxidant peptide from the skin of tropical frogs. Free Radic. Biol. Med..

[44] Yang H., Wang X., Liu X., Wu J., Liu C., Gong W., Zhao Z., Hong J., Lin D., Wang Y. (2009). Antioxidant peptidomics reveals novel skin antioxidant system. Mol. Cell. Proteom..

[45] Guo C., Hu Y., Li J., Liu Y., Li S., Yan K., Wang X., Liu J., Wang H. (2014). Identification of multiple peptides with antioxidant and antimicrobial activities from skin and its secretions of *Hylarana taipehensis*, *Amolops lifanensis*, and *Amolops granulosus*. Biochimie.

[46] Wang X., Ren S., Guo C., Zhang W., Zhang X., Zhang B., Li S., Ren J., Hu Y., Wang H. (2017). Identification and functional analyses of novel antioxidant peptides and antimicrobial peptides from skin secretions of four East Asian frog species. Acta Biochim. Biophys. Sin..

[47] Song Y.M., Yang S.T., Lim S.S., Kim Y., Hahm K.S., Kim J.I., Shin S.Y. (2004). Effects of L- or D-Pro incorporation into hydrophobic or hydrophilic helix face of amphipathic α-helical model peptide on structure and cell selectivity. Biochem. Biophys. Res. Commun..

[48] Manzo G., Scorciapino M.A., Srinivasan D., Attoub S., Mangoni M.L., Rinaldi A.C., Casu M., Flatt P.R., Conlon J.M. (2015). Conformational analysis of the host-defense peptides pseudhymenochirin-1Pb and -2Pa and design of analogues with insulin-releasing activities and reduced toxicities. J. Nat. Prod..

[49] Mangoni M.L., Di Grazia A., Cappiello F., Casciaro B., Luca V. (2016). Naturally occurring peptides from *Rana temporaria*: Antimicrobial properties and more. Curr. Top. Med. Chem..

[50] Irazazabal L.N., Porto W.F., Ribeiro S.M., Casale S., Humblot V., Ladram A., Franco O.L. (2016). Selective amino acid substitution reduces cytotoxicity of the antimicrobial peptide mastoparan. Biochim. Biophys. Acta.

[51] Iwamuro S., Kobayashi T., Soloviev M. (2010). An efficient protocol for DNA amplification of multiple amphibian skin antimicrobial peptide cDNAs. Peptidomics.

[52] Baik J.E., Choe H.I., Hong S.W., Kang S.S., Ahn K.B., Cho K., Yun C.H., Han S.H. (2016). Human salivary proteins with affinity to lipoteichoic acid of *Enterococcus faecalis*. Mol Immunol..

[53] Yamauchi R., Fukamizu S., Kohama Y., Shimamura T., Kashiwagi T., Ukeda H., Akiyama H., Mastui T., Ishikawa H. (2014). Comparative DPPH and ABTS radical scavenging activity assays for evaluating natural antioxidants as food additives. Food Preserv. Sci..

