# Supplementary figures and images for: Antimicrobial Property and Mode of Action of the Skin Peptides of the Sado Wrinkled Frog, Glandirana susurra, against Animal and Plant Pathogens
Source: Antibiotics (Basel). 2020 Jul 29;9(8):457. doi: 10.3390/antibiotics9080457 (PMC7460468; doi:10.3390/antibiotics9080457)

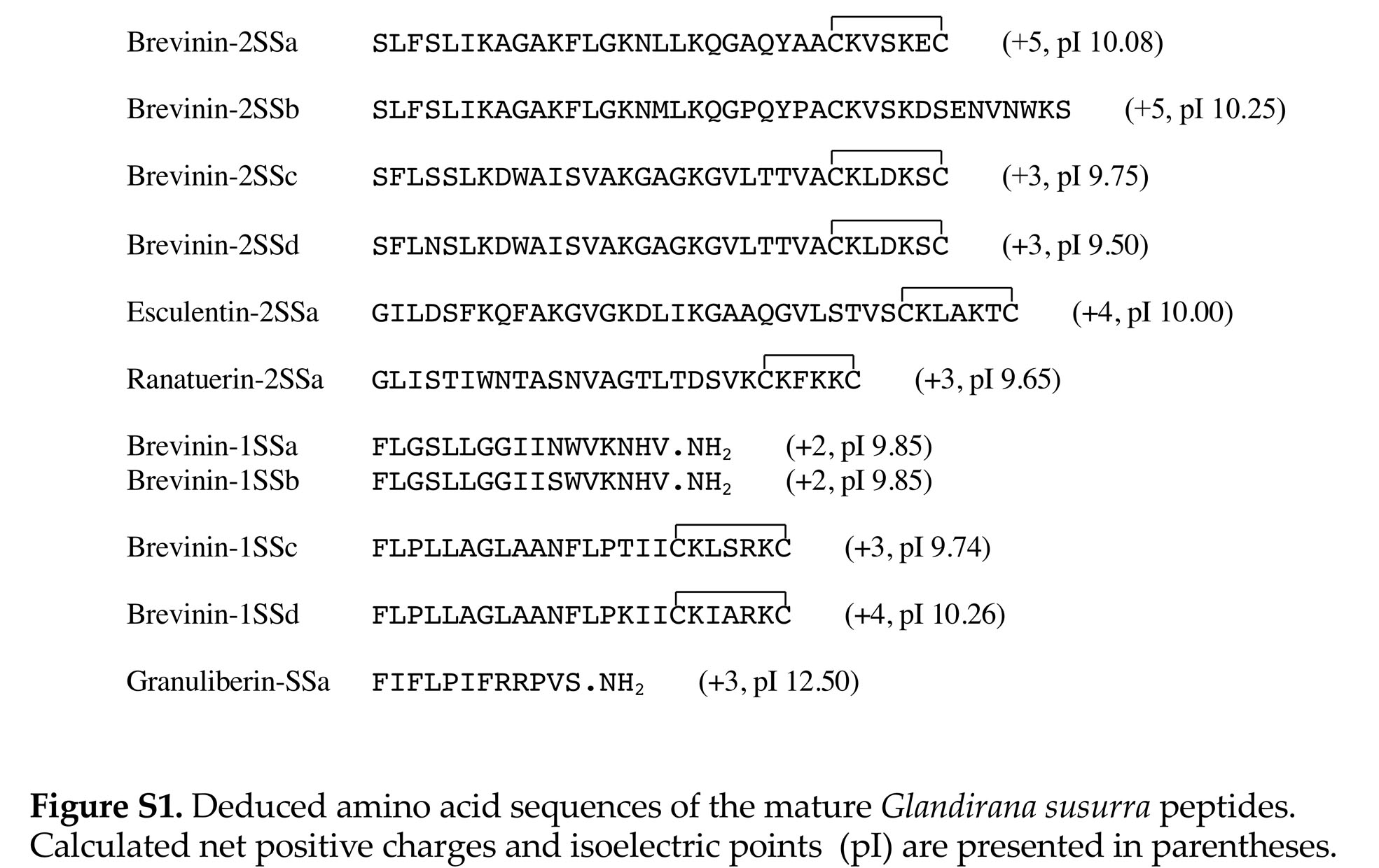

Supplement: Supplementary file 1 [file antibiotics-09-00457-s001.zip › DO.Antibiotics.rev.Fig.S1.jpg]

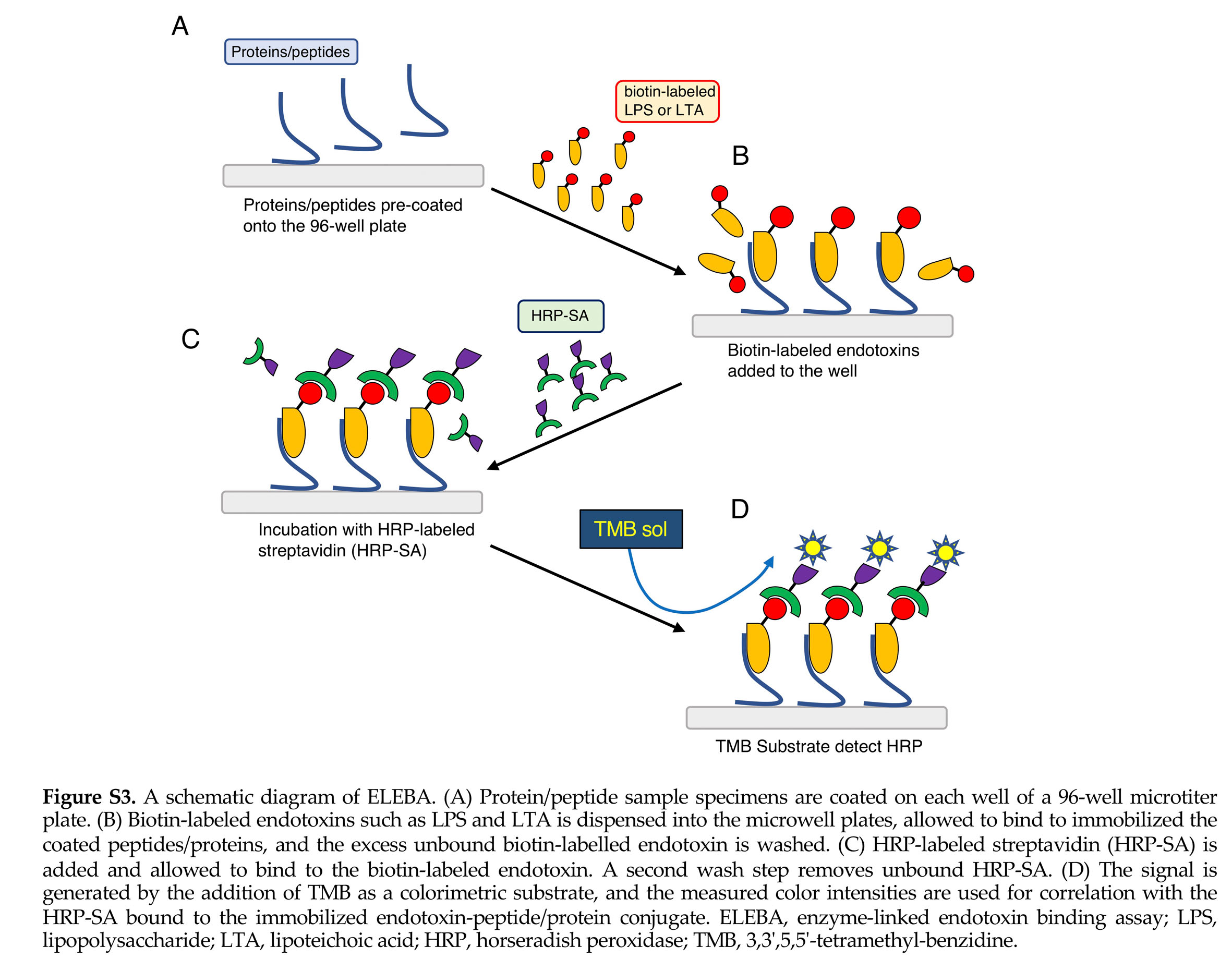

Supplement: Supplementary file 1 [file antibiotics-09-00457-s001.zip › DO.Antibiotics.rev.Fig.S3.jpg]

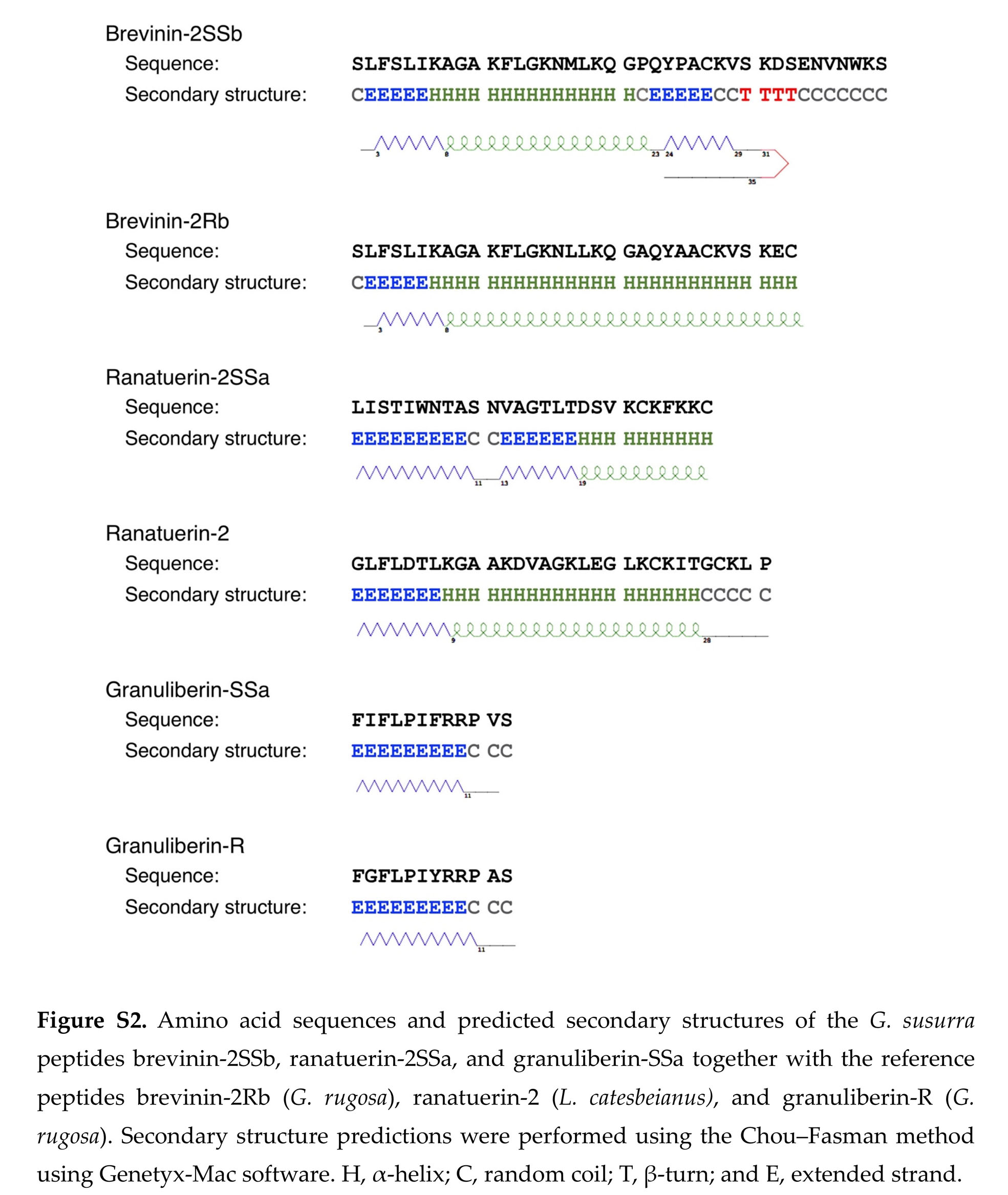

Supplement: Supplementary file 1 [file antibiotics-09-00457-s001.zip › DO.Antibiotics.revFig.S2.jpg]
